# Development of Genus-Specific PCR Primers for Molecular Monitoring of Invasive Nostocalean Cyanobacteria

**DOI:** 10.3390/ijerph18115703

**Published:** 2021-05-26

**Authors:** In-Soo Kim, Hae-Kyung Park, Yong-Jin Kim

**Affiliations:** Nakdong River Environment Research Center, National Institute of Environmental Research, Daegu 43008, Korea; factork@korea.kr (I.-S.K.); yjkim99@korea.kr (Y.-J.K.)

**Keywords:** invasive nostocalean cyanobacteria, *Cuspidothrix*, *Sphaerospermopsis*, *Cylindrospermopsis*, *Raphidiopsis*, *Chrysosporum*, genus-specific PCR primers

## Abstract

The geographical range of invasive cyanobacteria with high toxigenic potential is widening because of eutrophication and global warming, thus, monitoring their appearance is necessary for safe water quality control. Most invasive cyanobacteria are nostocalean species, and their accurate identification by classical morphological methods may be problematic. In this study, we developed polymerase chain reaction (PCR) primers to selectively identify five invasive cyanobacterial genera, namely, *Chrysosporum*, *Cuspidothrix*, *Cylindrospermopsis*, *Raphidiopsis*, and *Sphaerospermopsis*, using genetic markers such as *rbcLX*, *rpoB*, *rpoC1*, and *cpcBA*, and determined the amplification conditions for each pair of primers. The primer performances were verified on single or mixed nostocalean cyanobacterial isolates. The five primers allowed selective identification of all the target genera. In field samples collected during summer, when cyanobacteria flourished in the Nakdong River, the respective PCR product was observed in all samples where the target genus was detected by microscopic analysis. Besides, weak bands corresponding to *Sphaerospermopsis* and *Raphidiopsis* were observed in some samples in which these genera were not detected by microscopy, suggesting that the cell densities were below the detection limit of the microscopic method used. Thus, the genus-specific primers developed in this study enable molecular monitoring to supplement the current microscopy-based monitoring.

## 1. Introduction

Anthropogenic freshwater eutrophication causes harmful cyanobacterial blooms in streams and lakes worldwide. With the air and water temperatures increasing because of global warming and climate changes causing sudden intense rainfalls or increased water shortage, the intensity and duration of harmful cyanobacterial blooms are expected to increase. Moreover, these environmental changes extend the geographical range of invasive cyanobacteria from their original to new habitats [1,2,3,4]. Notably, most invasive cyanobacteria can produce toxins and gain a competitive advantage over native species, thus being regarded as a severe threat to aquatic ecosystems [2,5]. Therefore, monitoring potentially toxic invasive cyanobacteria is becoming an integral part of water quality control.

Members of the genera belonging to the order Nostocales of cyanobacteria have the advantages of producing dormant cells and fixing nitrogen. Some members of this order are *Cylindrospermopsis* spp., *Sphaerospermopsis* spp., *Cuspidothrix* spp., and *Chrysosporum* spp., which have been reported to invade new habitats in various environments [2,6,7,8]. These invasive nostocalean cyanobacteria are filamentous organisms, which have some overlapping morphological characteristics with each other and with some non-invasive species. Moreover, they exhibit huge morphological plasticity, and thus are difficult to identify. If cell density is low, the possibility of locating differentiated cells (akinetes and heterocytes)—being key features in identification—is low, leading to difficulties in the identification of cyanobacteria with high accuracy. Therefore, a more accurate and sensitive method than the existing microscopic analysis is needed for the monitoring of invasive nostocalean cyanobacteria.

Molecular phylogenetic systematic research has been conducted utilizing various genome sequences of cyanobacteria. In particular, genes encoding 16S/23S rRNA, the internal transcribed spacer (ITS), ribulose 1,5-bisphosphate carboxylase/oxygenase (Rubisco) large subunit (*rbcL*), nitrogen fixation (*nif*) functions, RNA polymerase beta subunit (*rpoB*), gyrase beta subunit (*gyrB*), and polyphosphate kinase (*ppk*) have been investigated as systematic genome markers [9,10,11,12]. The 16S rRNA gene has been most widely used for the identification and phylogenetic analyses. In a previous study, the 16S rRNA, *rbcLX*, and *rpoB* genes were used to compare the results obtained by morphological classification and phylogenetic analysis of *Nostocales* [13]. Moreover, a study was conducted on the identification and filiation of *Aphanizomenon* spp. using the 16S rRNA, *rbcLX*, and *cpcBA* genes [14]. In another study, changes in hereditary characteristics of members of the genus *Anabaena* depending on their habitat were revealed using the 16S rRNA, *rbcLX*, and *rpoC1* genes [15]. The lineage differentiation of cyanobacteria was studied using the 16S rRNA, *rbcLX*, and *hetR* genes [16]. Three genera, *Sphaerospermopsis*, *Cuspidothrix*, and *Chrysosporum*, were separated from the original genus *Aphanizomenon* based on the polyphasic approach, including ultrastructural and molecular characterization along with the conventional morphological classification [17]. Moreover, taxonomic delimitation of some freshwater planktonic nostocalean taxa, such as genus *Cylindrospermopsis* and *Raphidiopsis*, is still under debate [18].

In this study, as the first step in the molecular biological monitoring of invasive cyanobacteria of the order *Nostocales*, phylogeny and sequence similarities were analyzed to develop the genus-specific PCR primers using the housekeeping 16S rRNA, *rbcLX*, *rpoB*, *rpoC1*, and *cpcBA* genes. The polymerase chain reaction (PCR) primers developed in this study are expected to allow accurate early detection of potentially toxic invasive cyanobacteria and, consequently, enable their effective management.

## 2. Materials and Methods

### 2.1. Target Cyanobacteria

The target cyanobacteria included representatives of the typical invasive nostocalean genera *Cylindrospermopsis*, *Cuspidothrix*, *Chrysosporum*, *Sphaerospermopsis*, and *Raphidiopsis*. To obtain the target gene region of the target cyanobacteria, *Cylindrospermopsis* sp., *Cuspidothrix* sp., *Sphaerospermopsis* spp., and *Raphidiopsis* sp. were isolated from the Nakdong River in Korea and maintained in monocultures [19]. *Chrysosporum* spp. were not confirmed to appear in the Nakdong River, and therefore, *Chrysosporum ovalisporum* CS-1034 was obtained from the Australian National Algae Culture Collection. As a positive control, *Cuspidothrix issatschenkoi* NIVA CYA-711 was obtained from the Norwegian Culture Collection of Algae. *Raphidiopsis mediterranea* CS-1037 and *Cylindrospermopsis raciborskii* CS-1101 were obtained from the Australian National Algae Culture Collection (Table 1a). All strains were subcultured in MLA broth at a 2-week interval [20]. For genomic DNA extraction, each culture was proliferated until the late exponential phase and then filtered through a 0.45-μm MicronSep nitrocellulose membrane disk (GVS Life Sciences, Findlay, OH, USA). The filtered cyanobacteria on the membrane disk were stored in a −80 °C freezer until DNA extraction.

### 2.2. Sampling and Phytoplankton Analysis

To verify the applicability of the developed genus-specific primers for molecular monitoring using field samples, surface water was collected 500 m upstream of each of seven weirs on the Nakdong River from July to September 2019, when cyanobacterial proliferation was vigorous [21]. A total of 50 mL of water was filtered through a 0.45-μm MicronSep nitrocellulose membrane disk (GVS Life Sciences, Findlay, OH, USA), and the filtered sample on the disk was stored in a cryogenic freezer (−80 °C) until DNA analysis. For phytoplankton analysis, water samples were preserved by adding Lugol’s solution to a final concentration of 0.3%. Phytoplankton was classified to the genus or species level under a microscope (Imager M2, Carl Zeiss, Jena, Thüringen, Germany) [22,23]. Cells were counted using a Sedgwick–Rafter counting chamber, and the phytoplankton cell density was expressed as cells per milliliter of water.

### 2.3. Target Gene Sequences and Phylogenetic Analysis

#### 2.3.1. DNA Extraction

The DNeasy plant mini kit (Qiagen) was used for genomic DNA extraction from cultured cyanobacteria and field samples according to the protocol provided with the kit. To increase the DNA extraction efficiency, the filtered sample stored at −80 °C was incubated at room temperature for 5 min, followed by the addition of lysis buffer. This mixture was sonicated twice for bursts of 10 s each, at a constant amplitude of 20% of 130watt using VCX 130PB (Sonic & Materials, Inc. Newtown, CT, USA). The concentration and purity of the DNA were measured using an Infinite M200 PRO microplate reader (Tecan Austria GmbH, Grödig, Austria). The extracted genomic DNA was stored at −20 °C.

#### 2.3.2. Gene Sequence Characteristics and Phylogenetic Analysis

To develop genus-specific primers, the sequences of housekeeping genetic markers from *Aphanizomenon*, *Cuspidothrix*, *Cylindrospermopsis*, *Sphaerospermopsis*, and *Raphidiopsis* species were collected from the GenBank database registered in the US NCBI (National Center for Biotechnology Information, NIH, Bethesda, MD, USA) and all sequence information registered at the time of this study was used; 147 sequences of 16S rRNA, 140 sequences of *rbcLX*, 133 sequences of *cpcBA*, 134 sequences of *rpoC1*, and 65 sequences of *rpoB*. The DNA sequence similarity and genetic distances of target genes within nostocalean cyanobacteria were analyzed. Gene segments with the same base sequences were used for common sequences in the same species to eliminate sequence variations such as a single nucleotide polymorphism (SNP) within strains. DNA sequence similarity and genetic distance analysis was conducted after cutting off both ends of the aligned sequences, and the Kimura two-parameter model [24] was used in MEGA 6.0. Additionally, parsimony analysis (conserved sites, variable sites, and parsimony-informative sites) was carried out using MEGA 6.0. The DNA sequence similarity was expressed in percentage (%). The total average of sequence similarity was calculated by all sequence similarity values between species and expressed with standard deviation, and the number of samples (n) is the number of sequence similarities used in the calculation of the total average.

Phylogenetic analyses of target gene regions of the nostocalean genera were conducted using MEGA v.6 [24] and ClustalX2 [25], including sequences obtained from GenBank. The maximum-likelihood (ML) analysis of five regions was conducted using the RAxML 7.0.3 program with the default GTRGAMMA model [26]. Further, 200 independent tree inferences were used to identify the best tree. ML bootstrap values were determined using 1000 replicates, and Bayesian analyses were run using MrBayes v.3.1 [27] with the default GTR+G+I model to determine the best available model for the data from each region. Four independent Markov chain Monte Carlo runs were performed simultaneously for all sequence regions until the average standard deviation of split frequencies dropped below 0.01. The trees were sampled every 1000 generations. To ensure likelihood convergence, the first 1000 trees were discarded as burn-in. *Microcystis aeruginosa* was used as the outgroup.

### 2.4. Design of Genus-Specific Primers and Determination of PCR Amplification Conditions

Based on the analysis of the collected sequences described in Section 2.3.2, specific primers were designed for a gene with a genus-specific sequence. The sequence was subjected to multiple sequence alignment using MEGA 6.0, and the genus-specific sequence was searched for at intervals of 2 °C, depending on the primer type, in the range of 51 to 59 °C under annealing conditions using Primer3 (http://bioinfo.ut.ee/primer3/ accessed on 20 February 2019). For the selection of genus-specific primers, amplification test by touchdown PCR was performed with annealing temperatures incrementally decreasing by −0.2 °C for each cycle from 62 °C to 50 °C. The characteristics and potential hairpin formation of the search-specific primers were verified using Oligo Calculator version 3.27. Optimization of the annealing temperatures for the selected primers was carried out by performing gradient PCR with annealing temperatures in the range of 50 °C to 62 °C. For amplification of the target gene region, a 20-μL reaction mixture was made by mixing 1 ng of genomic DNA, 10 pmol of each PCR primer, and 10 μL of AccuPower^®^ Taq PCR premix (Bioneer, Deajeon, Korea). PCR was performed at 95 °C for 5min, followed by 30 cycles at 95 °C for 30 s, 55–59 °C (annealing temperature depending on each primer pair) for 30 s, and 1 min at 72 °C, and finally 10 min at 72 °C.

### 2.5. Verification of the Genus-Specific Primers

To verify the developed primers, one to three strains were cultured for each of the seven nostocalean genera used in this study, including the five-target invasive nostocalean genera (Table 1a). Genomic DNA was extracted from each culture, and PCR for each primer pair was performed. Moreover, single-cultured strains of the seven genera were mixed in seven different combinations (Table 1b). DNA was extracted from each mixed sample, and PCR was conducted using the developed primers.

To confirm the developed PCR primers’ applicability to field samples, surface water was collected 500 m upstream of the Gumi weir on 1 June 2020. Seven cultured cyanobacterial strains were added at different combinations to the collected water, resulting in eight samples (Table 1c), and PCR was performed using the developed primers and genomic DNA extracted from each sample. Furthermore, to verify the applicability of the developed primers for molecular monitoring using field samples, DNA was extracted from 11 field samples in which target cyanobacteria were observed under a microscope and 2 samples in which they were not observed. These samples were collected 500 m upstream of each of the seven weirs (Sangju, SJ; Nakdan, ND; Gumi, GM; Chilgok, CG; Dalseong, DS; Hapcheon-Changnyeong, HC; Changnyeong-Haman, CH) on the Nakdong River [21] between July and September 2019. The PCR results obtained with the developed genus-specific primers were compared with those of the microscopic examination.

To compare the specificity of the PCR primer pair developed in this study to genus *Cylindrospermopsis*, *Cylindrospermopsis*-specific PCR primer pair (*cyl2-cyl4*) developed by Wilson et al. [28] was used for PCR amplification.

Five target cyanobacterial strains were used as a positive control (Table 1a), and sterilized distilled water was used as a negative control. A 1.5% agarose gel was used for electrophoresis, and SiZer-100 (iNtRon, Seongnam, Korea) was used as a DNA marker to determine the sizes of the PCR products.

## 3. Results

### 3.1. Selection of Genetic Markers through Phylogenetic Analyses

In this study, to design PCR primers that could selectively distinguish the typical invasive nostocalean cyanobacterial genera, the 16S rRNA, *rbcLX*, *rpoB*, *rpoC1*, and *cpcBA* genes were used to identify the generic specificity of the target genes through phylogenetic classification.

**Figure 1:**
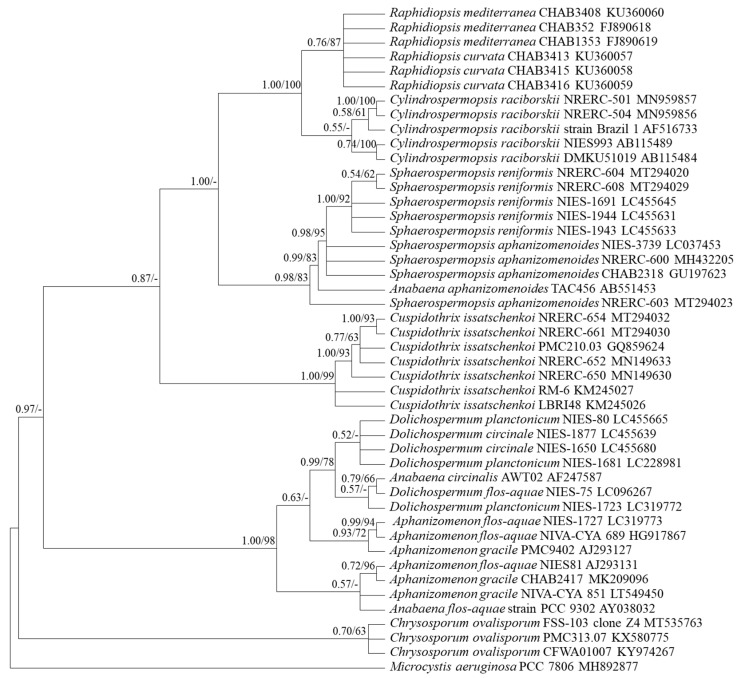
Consensus Bayesian tree based on aligned 1006-bp sequences of the 16S rRNA using the GTR+G+I model with *Microcystis aeruginosa* as an outgroup. The parameters were as follows: assumed equal nucleotide frequency; substitution rate matrix with A–C substitutions = 0.0928, A–G = 0.2562, A–T = 0.0772, C–G = 0.0801, C–T = 0.4256, and G–T = 0.0678; proportion of sites assumed to be invariable = 0.5723; and rates for variable sites assumed to follow a gamma distribution with the shape parameter = 0.2774. The branch lengths are proportional to the amount of character changes. The numbers above the branches indicate the Bayesian posterior probability (**left**) and maximum-likelihood bootstrap values (**right**). Posterior probabilities ≥ 0.5 are shown.

First, as the 16S rRNA was used as a conventional genomic marker for the classification of many cyanobacteria [29], phylogenetic analysis was performed using longer than 1006 bps of the 16S rRNA of nostocalean cyanobacterial species obtained from the NCBI. Based on the results, the genera *Sphaerospermopsis*, *Cuspidothrix*, and *Chrysosporum* of the target cyanobacteria formed independent clades, and the genera *Raphidiopsis* and *Cylindrospermopsis* formed one clade with separate branches (Figure 1).

**Figure 2:**
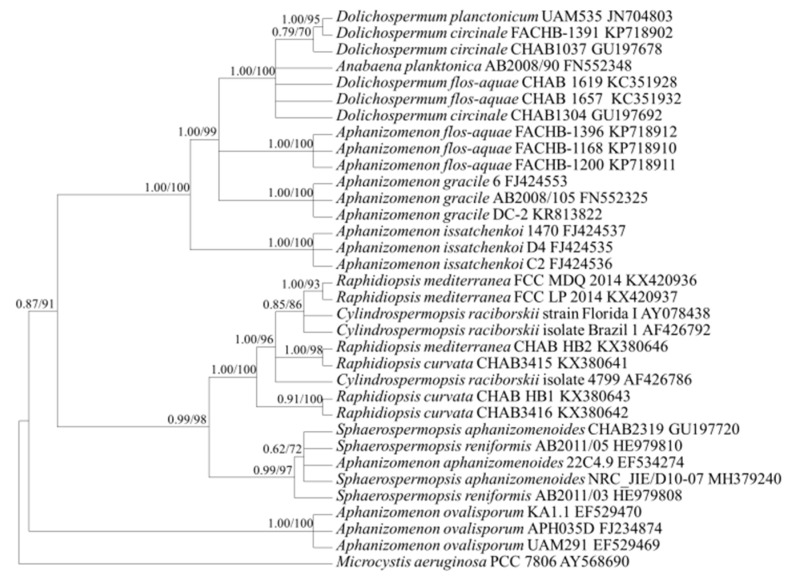
Consensus Bayesian tree based on aligned 322-bp sequences of *cpcBA* using the GTR+G+I model with *Microcystis aeruginosa* as an outgroup. The parameters were as follows: assumed equal nucleotide frequency; substitution rate matrix with A–C substitutions = 0.1590, A–G = 0.2297, A–T = 0.1304, C–G = 0.0518, C–T = 0.3314, and G–T = 0.0974; proportion of sites assumed to be invariable = 0.1173; and rates for variable sites assumed to follow a gamma distribution with the shape parameter = 6.8674. The branch lengths are proportional to the amount of character changes. The numbers above the branches indicate the Bayesian posterior probability (**left**) and maximum-likelihood bootstrap values (**right**). Posterior probabilities ≥ 0.5 are shown.

**Figure 3:**
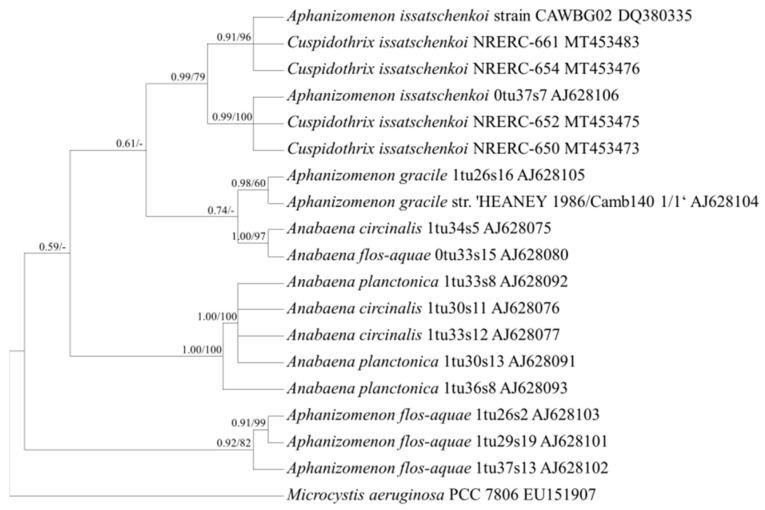
Consensus Bayesian tree based on aligned 499-bp sequences of *rpoB* using the GTR+G+I model with *Microcystis aeruginosa* as an outgroup. The parameters were as follows: assumed equal nucleotide frequency; substitution rate matrix with A–C substitutions = 0.0645, A–G = 0.2786, A–T = 0.0524, C–G = 0.1046, C–T = 0.4546, and G–T = 0.0451; proportion of sites assumed to be invariable = 0.2717; and rates for variable sites assumed to follow a gamma distribution with the shape parameter = 0.9189. The branch lengths are proportional to the amount of character changes. The numbers above the branches indicate the Bayesian posterior probability (**left**) and maximum-likelihood bootstrap values (**right**). Posterior probabilities ≥ 0.5 are shown.

**Figure 4:**
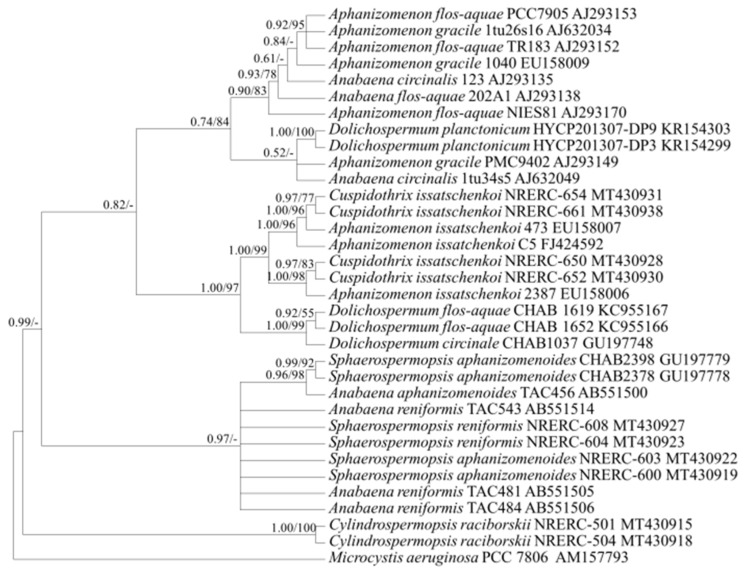
Consensus Bayesian tree based on aligned 993-bp sequences of *rbcLX* using the GTR+G+I model with *Microcystis aeruginosa* as an outgroup. The parameters were as follows: assumed equal nucleotide frequency; substitution rate matrix with A–C substitutions = 0.1028, A–G = 0.2947, A–T = 0.1220, C–G = 0.0857, C–T = 0.3330, and G–T = 0.0615; proportion of sites assumed to be invariable = 0.0155; and rates for variable sites assumed to follow a gamma distribution with the shape parameter = 0.9935. The branch lengths are proportional to the amount of character changes. The numbers above the branches indicate the Bayesian posterior probability (**left**) and maximum-likelihood bootstrap values (**right**). Posterior probabilities ≥ 0.5 are shown.

**Figure 5:**
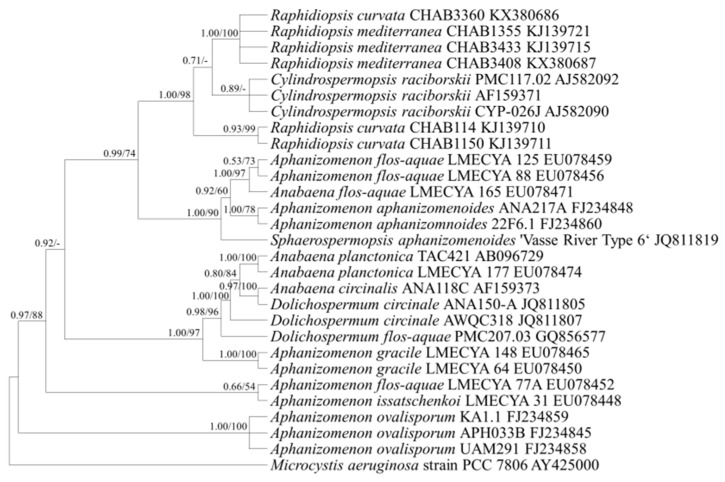
Consensus Bayesian tree based on aligned 373-bp sequences of *rpoC1* using the GTR+G+I model with *Microcystis aeruginosa* as an outgroup. The parameters were as follows: assumed equal nucleotide frequency; substitution rate matrix with A–C substitutions = 0.0455, A–G = 0.4384, A–T = 0.0295, C–G = 0.0844, C–T = 0.3488, and G–T = 0.0531; proportion of sites assumed to be invariable = 0.4420; and rates for variable sites assumed to follow a gamma distribution with the shape parameter = 1.1548. The branch lengths are proportional to the amount of character changes. The numbers above the branches indicate the Bayesian posterior probability (**left**) and maximum-likelihood bootstrap values (**right**). Posterior probabilities ≥ 0.5 are shown.

Based on the phylogenetic analysis using the *cpcBA* and *rpoB* gene sequences (Figure 2 and Figure 3), *C. issatschenkoi* (synonyms; *Anabaena issatschenkoi*, *Aphanizomenon issatschenkoi*) formed an independent clade. *C. issatschenkoi* also formed one clade with *Dolichospermum* spp., split into two branches based on the phylogenetic analysis using the *rbcLX* sequences (Figure 4), and one clade with *A. flos-aquae* based on the phylogenetic analysis using aligned 373-bp sequences of the *rpoC1* gene (Figure 5).

Based on the phylogenetic analysis using the *rpoC1* gene, the genera *Raphidiopsis* and *Cylindrospermopsis* formed separate branches within the same clade (Figure 5). The genera *Raphidiopsis* and *Cylindrospermopsis* also fell under the same clade and were not distinct based on the phylogenetic analysis using the *cpcBA* gene (Figure 2).

The genus *Chrysosporum* formed an independent clade distinguishable from the others by phylogenetic analysis using the *cpcBA* and *rpoC1* gene sequences (Figure 2 and Figure 5).

### 3.2. Design of Genus-Specific PCR Primers

Sequence similarity analysis of the 16S rRNA gene showed high average similarity among the genera (95.1 ± 2.6%; *n* = 66), and *Sphaerospermopsis*, *Cuspidothrix*, *Chrysosporum*, and *Raphidiopsis* showed a similarity of 94.6% with other cyanobacteria (Table 2). Although the target genera were distinguished by phylogenetic analysis using the 16S rRNA gene sequences, genus-specific sequences could not be found because of the high sequence similarity among the genera. Thus, this gene was excluded from designing genus-specific primers.

Based on the sequence similarity of the *rbcLX* gene, *Sphaerospermopsis aphanizomenoides* (synonyms; *Anabaena aphanizomenoides, Aphanizomenon aphanizomenoides*) showed a similarity of 83.4% with the other nostocalean cyanobacteria, which was lower than the total average sequence similarity of 88.3 ± 5.5% (*n* = 28), and the *cpcBA* gene was 74.6% similar to those of other compared taxa, which was close to the total average sequence similarity of 74.4 ± 10.2% (*n* = 45) (Table 2). For the *rbcLX* gene, with a lower similarity in *Sphaerospermopsis* spp. than the total average sequence similarity, a characteristic sequence with clear differences of 18 bp (forward) and 25 bp (reverse) in *Sphaerospermopsis* spp. was selected by a comparative analysis of multiple sequences from the nostocalean cyanobacteria listed in the NCBI database (Figure S1). Consequently, *Sphaerospermopsis* genus-specific primers *Sph-rbcLX*-F/R were designed. The sequences of the primer pair, annealing temperature and PCR product size are described in Table 3.

Based on the similarity analysis of the *rpoB* sequences, *C. issatschenkoi* was 78.2% similar to the other nostocalean cyanobacteria, which was slightly higher than the total average sequence similarity of 78.0 ± 8.4% (*n* = 15). Based on the analysis of the *cpcBA*, *rbcLX*, and *rpoC1* gene sequences, *C. issatschenkoi* had a similarity of 77.5%, 86.8%, and 76.0%, respectively, to the other nostocalean cyanobacteria, which were slightly higher or lower than the total average sequence similarity of 74.4 ± 10.2% (*n* = 45), 88.3 ± 5.5% (*n* = 28), and 75.6 ± 6.7% (*n* = 55) (Table 2). As *C. issatschenkoi* formed an independent clade in the *rpoB*-based phylogenetic tree and the gene had a lower sequence similarity than that of the other genes, *rpoB* sequences of six species of nostocalean cyanobacteria were subjected to multiple-sequence comparative analysis. The results showed that *Cuspidothrix* spp. had a characteristic sequence with differences of 4 bp (forward) and 6 bp (reverse) from those in the other genera. Thus, *Cuspidothrix* genus-specific primers, *Cus-rpoB*-F/R, were designed using this segment (Table 3 and Figure S2).

The sequence similarities of the *rpoC1* and *cpcBA* genes of *R. mediterranea* to those of the other compared taxa were 66.4% and 71.7%, respectively, which was lower than the total averages (75.6% and 74.4%, respectively). However, these genes in *R. mediterranea* showed 96.4% and 98.1% sequence similarity, respectively, with those of *C. raciborskii*, which was in the same clade by phylogenetic analysis (Table 2). Multiple-sequence comparative analysis of the *rpoC1* gene, which showed a high sequence similarity (94.8%) between *R. mediterranea* and *C. raciborskii*, found a characteristic sequence with differences of 1 bp (forward) and 3 bp (reverse) between two genera. Based on these results, *Raphidiopsis* genus-specific primers, *Raphi-rpoC1*-F/R, were designed (Table 3 and Figure S3). Furthermore, *Cylindrospermopsis* genus-specific primers, *Cyl-rpoC1*-F/R, were designed using sequences in the same region (Table 3).

Sequence similarity analysis of *rpoC1* and *cpcBA* genes of *C. ovalisporum* (synonyms; *Anabaena ovalisporum*, *Aphanizomenon ovalisporum*) showed that the values (70.1% and 67.2%, respectively) were lower than the total average values (75.6% and 74.4%, respectively) (Table 2). Based on multiple-sequence comparative analysis of the *cpcBA* gene, with a lower sequence similarity, a characteristic sequence was selected, which showed differences of 6 bp (forward) and 8 bp (reverse) between *C. ovalisporum* and other species, and *Chrysosporum* genus-specific primers, *Chry-cpcBA*-F/R, were designed (Table 3 and Figure S4).

### 3.3. Verification of the Genus-Specific Primers

**Figure 6:**
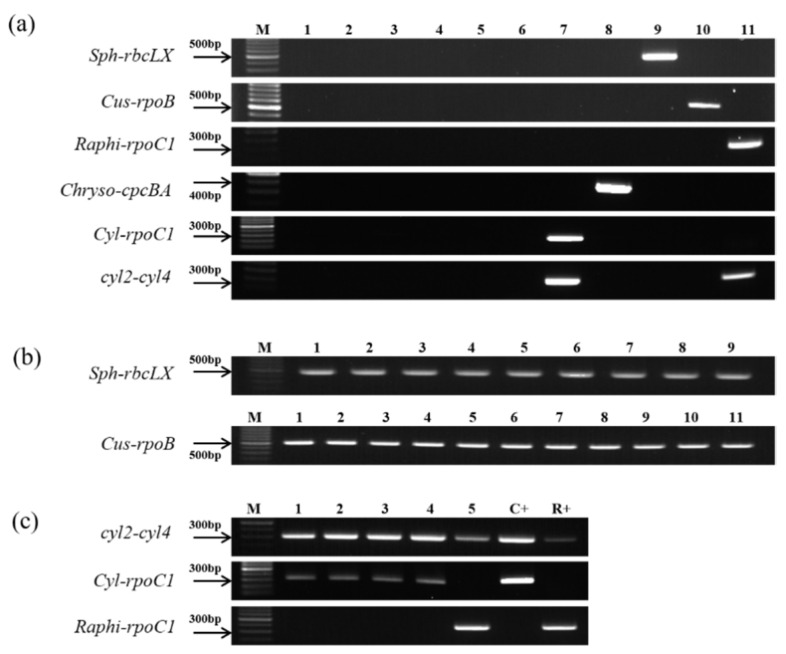
Agarose gel electrophoresis of polymerase chain reaction (PCR) products obtained with genus-specific primers. (**a**) PCR products from cultured nostocalean cyanobacterial strains (please refer to Table 1a for numbering). (**b**) PCR products from (upper panel) *Sphaerospermopsis aphanizomenoides* NRERC-600–603 (1–4) and 605–607 (6–8); *Sphaerospermopsis reniformis* NRERC-604 and 608 (5 and 9); and (lower panel) *Cuspidothrix issatschenkoi* NRERC-650–652 and 654–661 (1–11) isolated from the Nakdong River. (**c**) PCR products from *Cylindrospermopsis* sp. NRERC-501–504 (1–4) and *Raphidiopsis curvata* NRERC-701 (5) isolated from the Nakdong River. M: SiZer-100 DNA marker.

DNA from morphologically and genetically similar members of the seven nostocalean cyanobacterial genera (Table 1a), including the five target genera, was amplified using the developed primers. The results showed that each primer pair amplified a PCR product only for members of the target genus, while no bands were observed for other genera members (Figure 6a). Therefore, it was confirmed that the five pairs of PCR primers designed in this study specifically reacted with the DNA of each target species from the various nostocalean cyanobacterial genera.

Furthermore, DNA was amplified from strains that were isolated from the Nakdong River and identified based on their morphological characteristics. The *Cuspidothrix* genus-specific primers *Cus-rpoB*-F/R were used with DNA from 11 strains of *C. issatschenkoi* (NRERC-650–652 and 654–661), while the *Sphaerospermopsis* genus-specific primers *Sph-rbcLX*-F/R were used with DNA from 7 strains of *S. aphanizomenoides* (NRERC-600–603 and 605–607) and 2 strains of *Sphaerospermopsis reniformis* (basionym; *Anabaena reniformis*) (NRERC-604 and 608). The results confirmed the amplification of PCR products of the correct sizes for all isolates (Figure 6b), showing consistency with morphological identification data.

With the *Raphi-rpoC1*-F/R primer pair, a PCR product was only observed for *R. mediterranea* CS-1037 but not for other nostocalean strains, including members of the genus *Cylindrospermopsis* (Figure 6a). This result suggests that this primer pair specifically targets the DNA of *Raphidiopsis* spp. but not that of *Cylindrospermopsis* spp., which show high sequence similarity; thus, this primer pair can be used to distinguish members of the two genera in natural water. Meanwhile, after PCR amplification of the same samples using the *Cylindrospermopsis*-specific PCR primer (*cyl2-cyl4*) developed by Wilson et al. [28], PCR products were obtained for members of both *Cylindrospermopsis* and *Raphidiopsis*, suggesting that the two genera could not be differentiated (Figure 6a). However, after PCR amplification using the *Cyl-rpoC1*-F/R primers developed in this study, a PCR product was only observed in the *Cylindrospermopsis* sp. sample but not in that of *Raphidiopsis* sp. (Figure 6a). Moreover, PCR amplification of DNA from four strains of *C. raciborskii* (NRERC-501–504) and one strain of *R. curvata* (NRERC-701), which were isolated from the Nakdong River, was only positive for the respective targets when using the primers *Raphi-rpoC1*-F/R and *Cyl-rpoC1*-F/R; however, PCR products were observed for the members of both genera when using the *cyl2-cyl4* primers (Figure 6c). Therefore, it is expected that the morphologically and genetically similar genera *Raphidiopsis* and *Cylindrospermopsis* can be effectively distinguished using the *Raphi-rpoC1*-F/R and *Cyl-rpoC1*-F/R primers developed in this study.

**Figure 7:**
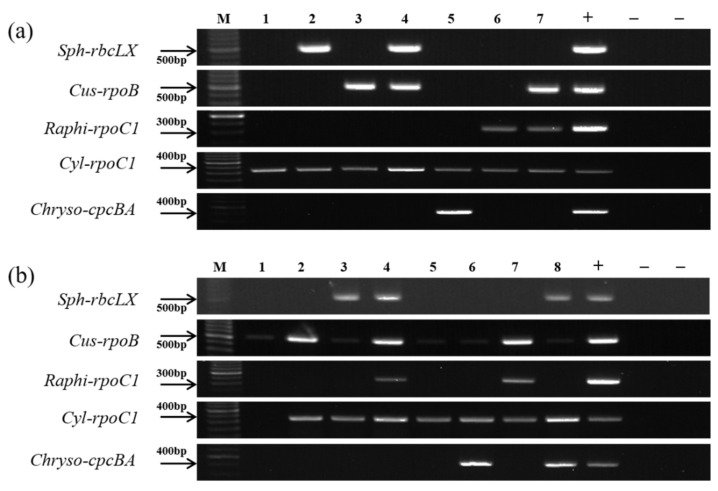
Agarose gel electrophoresis of PCR products obtained with genus-specific primers in (**a**) mixed samples of various cyanobacterial cultures (Table 1b) and (**b**) field samples mixed with cyanobacterial cultures (Table 1c). Positive (+) and negative (−) controls were used as described in the Materials and Methods. M: SiZer-100 DNA marker.

To verify whether the five developed PCR primer pairs could act in a genus-specific manner in mixed samples of multiple nostocalean cyanobacteria, PCR was performed on samples of randomly mixed cultured strains (Table 1b). All five primer pairs only amplified PCR products in the target-positive controls but did not react with DNA from other nostocalean cyanobacteria, confirming that the five pairs of primers could accurately distinguish the target cyanobacteria (Figure 7a).

To investigate the deterrent effect due to interfering substances, such as algae (bacillariophytes, green algae, flagellates, etc.) and organic materials, that could affect the reaction of the five developed PCR primer pairs, cultures of the seven nostocalean cyanobacterial genera were added to the surface water collected from the Nakdong River (spike), and the reaction status of the primers was verified. Based on the microscopic analysis of the phytoplankton in the surface water collected at the Gumi weir section on 1 June 2020, the cell density of the total phytoplankton was 26,344 cells·mL^−1^, including 17,080 cells·mL^−1^ of bacillariophytes, 2100 cells·mL^−1^ of green algae, 2920 cells·mL^−1^ of flagellates, and 4244 cells·mL^−1^ of cyanobacteria. The most dominant genus among the cyanobacteria was *Pseudanabaena*, and the cell density of nostocalean cyanobacteria was 1164 cells·mL^−1^, with 711 cells·mL^−1^ of *C. issatschenkoi* and no other target cyanobacteria from this study. The field samples without added culture samples only generated PCR products with the *Cus-rpoB* primers but not with the other primers. For the seven field samples with different combinations of cultured cyanobacterial strains (Table 1c), PCR products were obtained with each primer pair when target cyanobacteria were added (Figure 7b). Based on these results, the primers developed in this study can successfully distinguish the target cyanobacteria even when other cyanobacteria and non-cyanobacterial organisms are mixed with the target cyanobacteria.

**Figure 8:**
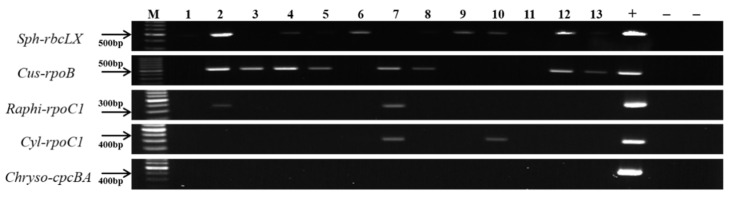
Agarose gel electrophoresis of PCR products obtained with the genus-specific primers on samples collected from 13 points on the Nakdong River. Positive (+) and negative (−) controls were as described in the Materials and Methods. M: SiZer-100 DNA marker.

To examine the field applicability of the PCR primers developed in this study, the reaction status of the primers was investigated with the river water. The phytoplankton population analysis was carried out using a microscope on 13 surface water samples collected 500 m upstream of each of seven weirs on the Nakdong River from July to September 2019, the summer season when cyanobacteria flourished in the Nakdong River. The results indicated that bacillariophytes (*Aulacoseira* spp. and *Skeletonema* spp.) and cryptophytes (*Cryptomonas* spp.) were dominant in two (Nos. 10 and 13) and one sample (No. 8), respectively. Cyanobacteria dominated the remaining ten samples. *Microcystis* spp., *Dolichospermum* spp., and *Merismopedia* spp. were dominant in seven (Nos. 2, 4, 6, 8, 9, 11 and 12), one (No. 3), and two (Nos. 1 and 5) samples, respectively. Among the cyanobacteria targeted by the PCR primers developed in this study, *Cuspidothrix* spp., *Cylindrospermopsis* spp., and *Sphaerospermopsis* spp. were observed in eight, two, and six samples, respectively, while *Chrysosporum* spp. and *Raphidiopsis* spp. were not observed in any of the 13 samples (Table 4). According to the microscopic analysis, after amplifying the 13 samples with the *Cuspidothrix*-specific primers, PCR products were detected in all *Cuspidothrix*-positive samples, but not in other samples. For *Chrysosporum* spp., consistent results were obtained by microscopy and gene analysis, with no PCR products detected in the 13 samples. For *Sphaerospermopsis* spp., PCR products were found in the six samples confirmed by microscopy, and a weak electrophoretic band was observed in another sample (No. 13). *Raphidiopsis* spp. were not observed by microscopic analysis, but a weak band was detected by gene analysis (Nos. 2 and 7) (Figure 8).

## 4. Discussion

The housekeeping genetic markers used in this study are all genes widely utilized in various phylogenetic studies of cyanobacteria to compensate for the limitation of 16S rRNA. The *rbcLX* sequence contains *rbcL* and *rbcX*, the genes encoding two subunits of the RuBisCO (ribulose-bisphosphate carboxylase), separated by an intergenic spacer (IGS). *rbcLX* was used to bypass the limitations of *rbcL*, which had been conventionally widely utilized and was first used to establish the phylogenetic relationship between the genera *Dolichospermum* (formerly *Anabaena*) and *Aphanizomenon* by Gugger [10]. The *rpoB* and *rpoC1* genes encode the beta and gamma subunits of RNA polymerase, respectively, and were used in previous studies for lineage differentiation, including a study on the evolutionary association of chloroplasts of eubacteria and cyanobacteria [30]. *rpoB* was first used to supplement the limitations of the 16S rRNA in a study by Dalhllöf [9], and *rpoC1* was used, along with the 16S rRNA, *hetR*, and *nifH* genes, in a study of Thomazeau on the phylogeny of cyanobacteria in sub-Saharan Africa [31], which shows the low genetic diversity of *C. raciborskii* [32,33]. *cpcBA* includes sequences of the *cpcB* and *cpcA* genes, encoding for phycocyanin subunits, and an IGS. *cpcBA* was first used as a complementary gene for addressing the limitations of the 16S rRNA gene in a phylogeographical study of the genus *Synechococcus* [34]. *cpcBA* was also used as a geographical marker of the genus *Cylindrospermopsis* in a study by Dyble [35]. Phylogenetic analysis based on these genetic markers, including 16S rRNA, showed that the target nostocalean cyanobacterial genera in this study formed an independent clade from other genera, indicating the availability of these genetic markers in designing PCR primers that specifically distinguish target nostocalean cyanobacterial genera.

In the conventional morphological classification system, *Cylindrospermopsis* spp. and *Raphidiopsis* spp., which have a similar trichome shape, were distinguished by forming heterocytes. If conical heterocytes were formed at the end of the trichome, the organism was classified to the genus *Cylindrospermopsis*; if no heterocytes were formed, the organism was classified to the genus *Raphidiopsis* [22]. However, it has recently been shown that the genera *Raphidiopsis* and *Cylindrospermopsis* form a monophyletic lineage and are not distinguished [18]. Hence, it has been proposed that these two genera are combined in the genus *Raphidiopsis* based on a phylogenetic study that used the 16S rRNA gene, 16S–23S ITS, and *cpcBA* sequences [18]. Consequently, a few studies have reported *C. raciborskii* as *Raphidiopsis raciborskii* [36,37,38,39]. This study also showed that these two genera were not separated in the phylogenetic tree of 16S rRNA, *rpoC1*, and *cpcBA* genes (Figure 1, Figure 2 and Figure 5). However, the integration of the two genera has still been disputed, and many researchers have differentiated *Raphidiopsis* and *Cylindrospermopsis*. Accurate identification of *Cylindrospermopsis* spp. in the proliferation stage, when end cells do not appear to differentiate into heterocytes, is difficult in field samples. However, since *Cylindrospermopsis* spp. and *Raphidiopsis* spp. have a nucleotide sequence similarity of about 98.1% in the *rpoC1* gene, it is challenging to generate a primer with the conventional primer design method. It was not easy to find the nucleotide sequence section that distinguishes the *Raphidiopsis* spp. from other genera including *Cylindrospermopsis*, and even in the section where there is a difference between *Raphidiopsis* spp. and other genera, the nucleotide sequence different from *Cylindrospermopsis* did not exceed 3 bp in the entire section. Therefore, since there is only 1 bp difference in forward primer, it is impossible to distinguish between *Cylindrospermopsis* and *Raphidiopsis* spp., but in reverse primer, 1 and 2 bp different sequences are located at the 3′ terminus causing primer mismatch. This type of primer design has been reported in a study of Liu’s allele-specific primers for rapeseed (*Brassica napus L*) and sesame (*Sesamum indicum*) [40]. According to Liu’s study, the primer mismatch ratio according to the type and position of single nucleotide polymorphism (SNP) base pairs left at the 3′ terminus is different, and using this, allele-specific primer design is possible. The sequence difference between *Cylindrospermopsis* and *Raphidiopsis* spp. in the 1st and 4th to 5th position from the 3′ terminus of the reverse primer can cause a mismatch with a probability of 22.5%, 31.7% and 32.9%, respectively. As a result, it was possible to distinguish these two genera in the actual PCR amplification. This result signifies that a high overall similarity of genes does not prevent the primer design and the taxonomic distinction between the two genera. Furthermore, this primer design method can increase primer specificity and be applied in other cyanobacterial species with high genetic similarity.

It is assumed that the discrepancy between the microscopic results and molecular monitoring results in the field samples is due to the difference in the two methods’ detection limits. It is challenging to distinguish *S. aphanizomenoides* from *Aphanizomenon* spp. or *Dolichospermopsis* spp. if an akinete adjacent to a heterocyte is not found, while *Raphidiopsis* spp. are morphologically similar to *Cuspidothrix* spp. and *Cylindrospermopsis* spp., particularly when the trichomes are young. Therefore, for a trichome at its early stage of proliferation, morphological characteristics that serve as identification keys might not have appeared, and thus there is a possibility that it was falsely identified as *Aphanizomenon* spp. or *Cuspidothrix* spp., which appeared at relatively high densities. Moreover, we cannot exclude that *S. aphanizomenoides* and *Raphidiopsis* spp. appeared at a low density and could not have been detected by our protocol of microscopic analysis but were detected by PCR. In the filamentous cyanobacteria, a few or dozens of cells form one filament, and if the concentration is less than one filament per milliliter, no microscopic detection is registered if 1 mL of water is analyzed, and also more time-consuming methods such as the concentration of samples are needed to detect by microscopy. However, molecular methods such as PCR amplification allow the detection of even a single cell, thus enabling identification at a very low cell density. In this study, PCR products were detected in samples in which target cyanobacteria were not detected by microscopic analysis of 1 mL, whereas DNA was extracted after filtering a minimum of 50 mL of river water. Furthermore, the shape of special cells, such as heterocytes, akinetes, and apical cells, is used for morphological identification of most nostocalean cyanobacteria. However, if a cyanobacterium is present at low concentration, it is difficult to observe a specific cell shape and to identify the organism accurately. Therefore, the use of the PCR primers developed in this study is expected to allow the accurate identification of the target cyanobacteria present at a low concentration.

Although this study was initially aimed to develop species-specific primers, due to the limited sequence information in the NCBI database (number of sequences and differences among the regions sequenced) and a shortage of positive control strains, primers were developed for representative species in each target genus. Although the developed primers distinguished the target genera, the reaction status was not investigated for different species in the genera *Cuspidothrix* and *Cylindrospermopsis*. For the genus *Sphaerospermopsis*, after isolating *S. aphanizomenoides* and *S. reniformis* and applying the primers developed in this study, the PCR product was amplified from DNA of both species, suggesting primer specificity, not for *S. aphanizomenoides* but the genus *Sphaerospermopsis*. In the case of *Raphidiopsis* spp., the PCR product was generated not only for *R. mediterranea*, isolated in Australia, but also for *R. curvata*, isolated from the Nakdong River, indicating that the primer selectively targeted the genus *Raphidiopsis*. Therefore, the primers developed in this study were determined to be genus-specific primers that can distinguish the target genera. We plan to additionally confirm their genus-specific properties after securing genetic information and cultured strains for other species in the target genera.

Among the target species used in this study, *C. issatschenkoi* and *C. raciborskii* are toxic species that have been reported to produce anatoxin-a and cylindrospermopsin [5,19,36,41]. *Raphidiopsis brookii* has been reported to secrete saxitoxin [42], and *R. curvata* has been reported to secrete cylindrospermopsin [43]. Furthermore, it has been reported that *R. mediterranea* secretes anatoxin [44]. Although *Chrysosporum* spp. do not secrete saxitoxin, they carry the *sxtA* gene, suggesting their potential toxigenicity [41,45,46], and are known to secrete cylindrospermopsin [47,48]. *S. aphanizomenoides* has also been reported to have a potential toxin-producing capability [1,6,49,50]. Moreover, these species are invasive nostocalean cyanobacteria, reported to extend their habitation range; hence, continuous monitoring is required in water habitats where these algae have not yet appeared. However, it is difficult to detect their occurrence accurately with the existing microscopic analysis techniques when there are no or low-density differentiated cells. Primer pairs that can specifically distinguish the target genera of this study have not been reported in previous studies as far as the authors know, and were first developed through this study. Therefore, using the genus-specific primers developed in this study, molecular monitoring, which can provide more accurate identification of cyanobacteria and information on their appearance status, may supplement the existing microscopy-based monitoring and, consequently, allow proactive preparation for the potential cyanotoxin production in the environment.

## 5. Conclusions

In recent years, the habitats of invasive toxic nostocalean cyanobacteria are continuously expanding, so it is necessary to monitor their appearance in waters where they have never been found. In this study, we developed PCR primers that specifically identify and detect five common invasive nostocalean genera, *Chrysosporum*, *Cuspidothrix*, *Cylindrospermopsis*, *Raphidiopsis*, and *Sphaerospermopsis*. Five pairs of PCR primers based on housekeeping genetic markers could accurately detect the target cyanobacteria in the culture and field samples. These results are expected to enable the early detection of potentially toxic invasive cyanobacteria and their effective management. Moreover, these primers can distinguish two genera which have high overall genetic similarity. These results show the applicability of molecular monitoring for morphologically and genetically similar cyanobacterial genera using highly specific primer pairs. The developed primers could distinguish different genera; however, species-level identification could not be achieved because the necessary genetic information for each species is not currently available and because of the lack of corroborative positive control possessed. If these challenges are addressed in the future, primers that can more accurately distinguish different species could be developed.

## Figures and Tables

**Table 1:** List of cultured strains of cyanobacteria (**a**), mixed strain samples (**b**) and field samples (with or without cultured strains added) (**c**) used to test the applicability of developed genus-specific primers; ‘+’ represents addition of the cultured strains, ‘-’ represents no addition of the cultured strains.

**(a) Monocultures of strains.**																
**Sample No**	**Scientific Name**	**Strain No.**								**Positive Control**						
1	*Aphanizomenon flos-aquae*	NRERC-008								no						
2	*Aphanizomenon flos-aquae*	NRERC-009								no						
3	*Aphanizomenon gracile*	NH-5								no						
4	*Dolichospermum planctonicum*	NRERC-101								no						
5	*Dolichospermum circinale*	NRERC-103								no						
6	*Dolichospermum flos-aquae*	NRERC-108								no						
7	*Cylindrospermopsis raciborskii*	CS-1101								yes						
8	*Chrysosporum ovalisporum*	CS-1034								yes						
9	*Sphaerospermopsis aphanizomenoides*	NRERC-600								yes						
10	*Cuspidothrix issatschenkoi*	NIVA CYA-711								yes						
11	*Raphidiopsis mediterranea*	CS-1037								yes						
**(b) Mixed samples with different combinations of monocultures of strains.**																
**Sample No.** **Strains**				**1**		**2**		**3**			**4**		**5**	**6**		**7**
*Aphanizomenon flos-aquae* NRERC-008				+		+		+			+		+	+		+
*Aphanizomenon gracile* NH-5				+		+		+			+		+	+		+
*Dolichospermum planctonicum* NRERC-101				+		+		+			+		+	+		+
*Cylindrospermopsis raciborskii* CS-1101				+		+		+			+		+	+		+
*Sphaerospermopsis aphanizomenoides* NRERC-600				-		+		-			+		-	-		-
*Cuspidothrix issatschenkoi* NIVA CYA-711				-		-		+			+		-	-		+
*Raphidiopsis mediterranea* CS-1037				-		-		-			-		-	+		+
*Chrysosporum ovalisporum* CS-1034				-		-		-			-		+	-		-
**(c) Spiked field samples with different combinations of monocultures of strains.**																
**Sample No.** **Strains**			**1**		**2**		**3**		**4**			**5**	**6**		**7**	**8**
*Aphanizomenon gracile* NH-5			-		+		+		+			+	+		+	+
*Dolichospermum planctonicum* NRERC-101			-		+		+		+			+	+		+	+
*Cylindrospermopsis raciborskii* CS-1101			-		+		+		+			+	+		+	+
*Sphaerospermopsis aphanizomenoides* NRERC-600			-		-		+		+			-	-		-	+
*Cuspidothrix issatschenkoi* NIVA CYA-711			-		+		-		+			-	-		+	-
*Raphidiopsis mediterranea* CS-1037			-		-		-		+			-	-		+	-
*Chrysosporum ovalisporum* CS-1034			-		-		-		-			-	+		-	+

**Table 2:** Sequence similarities (%) of target genes (a; *16S rRNA*, b; *rbcLX*, c; *cpcBA*, d; *rpoB*, e; *rpoC*) among nostocalean cyanobacteria.

**(a) *16S rRNA* (1306 bp)**		**(1)**			**(2)**				**(3)**			**(4)**					**(5)**				**(6)**				**(7)**				**(8)**					**(9)**					**(10)**			**(11)**					**(12)**
(1) *Aphanizomenon flos-aquae*																																															
(2) *Aphanizomenon gracile*		98.7																																													
(3) *Dolichospermum planctonicum*		98.1			98.2																																										
(4) *Dolichospermum flos-aquae*		97.8			98.4				97.3																																						
(5) *Dolichospermum circinale*		98.6			98.4				99.3			97.5																																			
(6) *Cylindrospermopsis raciborskii*		93.3			93.6				93.5			93.1					93.7																														
(7) *Chrysosporum ovalisporum*		95.2			95.6				95.1			94.7					95.3				93.5																										
(8) *Sphaerospermopsis reniformis*		93.1			93.3				93.1			92.0					92.9				96.1				93.8																						
(9) *Sphaerospermopsis aphanizomenoides*		93.4			93.7				93.3			92.4					93.2				96.7				94.1				99.5																		
(10) *Cuspidothrix issatschenkoi*		95.6			96.7				95.2			96.1					95.0				93.4				94.6				93.7					94.1													
(11) *Raphidiopsis mediterranea*		93.4			93.7				93.6			93.1					93.8				99.7				93.5				96.4					96.9					93.5								
(12) *Raphidiopsis curvata*		93.1			93.7				93.3			93.0					93.4				99.3				93.0				96.0					96.5					93.1			99.2					
**(b) *rbcLX*** **(761 bp)**	(1)						(2)						(3)							(4)						(5)					(6)						(7)						(8)				
(1) *Aphanizomenon flos-aquae*																																															
(2) *Aphanizomenon gracile*	100.0																																														
(3) *Dolichospermum planctonicum*	94.9						94.9																																								
(4) *Dolichospermum flos-aquae*	89.0						89.0						90.9																																		
(5) *Dolichospermum circinale*	88.7						88.7						90.8							99.3																											
(6) *Sphaerospermopsis aphanizomenoides*	85.4						85.4						85.0							82.1						81.6																					
(7) *Sphaerospermopsis reniformis*	85.6						85.6						85.4							81.6						81.1					98.6																
(8) *Cuspidothrix issatschenkoi*	88.1						88.1						86.2							91.4						91.5					81.0						81.1										
**(c) *cpcBA*** **(357 bp)**				(1)				(2)						(3)				(4)				(5)					(6)					(7)			(8)					(9)					(10)		
(1) *Aphanizomenon flos-aquae*																																															
(2) *Aphanizomenon gracile*				90.0																																											
(3) *Dolichospermum planctonicum*				81.2				77.9																																							
(4) *Dolichospermum flos-aquae*				90.0				100.0						77.9																																	
(5) *Cylindrospermopsis raciborskii*				67.8				64.3						66.9				64.3																													
(6) *Chrysosporum ovalisporum*				66.4				63.5						66.4				63.5				68.3																									
(7) *Sphaerospermopsis aphanizomenoides*				74.8				72.9						75.2				72.9				77.2					70.9																				
(8) *Cuspidothrix issatschenkoi*				86.2				83.2						78.0				83.2				73.4					71.0					72.8															
(9) *Raphidiopsis mediterranea*				66.1				63.2						66.9				63.2				98.1					68.1					76.2			71.8												
(10) *Raphidiopsis curvata*				67.2				64.5						67.1				64.5				95.8					66.4					78.6			73.0					95.3							
**(d) *rpoB*** **(504 bp)**										(1)						(2)							(3)							(4)						(5)								(6)			
(1) *Aphanizomenon flos-aquae*																																															
(2) *Aphanizomenon gracile*										99.4																																					
(3) *Dolichospermum planctonicum*										80.5						79.9																															
(4) *Dolichospermum circinale*										74.8						74.8							85.8																								
(5) *Dolichospermum flos-aquae*										74.6						74.6							69.3							64.7																	
(6) *Cuspidothrix issatschenkoi*										84.5						84.2							77.5							69.6						75.1											
**(e) *rpoC1*** **(452 bp)**			(1)			(2)					(3)				(4)				(5)					(6)				(7)					(8)					(9)			(10)					(11)	
(1) *Aphanizomenon flos-aquae*																																															
(2) *Aphanizomenon gracile*			78.1																																												
(3) *Dolichospermum planctonicum*			77.2			81.2																																									
(4) *Dolichospermum flos-aquae*			75.4			74.6					72.9																																				
(5) *Dolichospermum circinale*			75.8			79.9					89.6				73.2																																
(6) *Cylindrospermopsis raciborskii*			75.1			71.0					71.6				69.7				71.2																												
(7) *Chrysosporum ovalisporum*			70.7			66.7					70.3				77.9				69.0					67.8																							
(8) *Sphaerospermopsis aphanizomenoides*			93.5			77.7					75.9				74.6				76.4					76.1				70.7																			
(9) *Cuspidothrix issatschenkoi*			79.8			79.5					76.3				74.8				78.4					73.5				71.9					80.5														
(10) *Raphidiopsis mediterranea*			73.4			70.4					71.7				69.5				69.6					96.4				66.0					74.5					72.8									
(11) *Raphidiopsis curvata*			76.5			73.2					74.4				70.8				72.0					94.2				69.6					77.1					72.5			93.7						

**Table 3:** Genus-specific primers developed in this study.

Target Genus	Marker	Primer	Sequence (5′→3′)	Annealing Temperature (°C)	Product Size (bp)
*Sphaerospermopsis*	*rbcLX*	*Sph-rbcLX–*F	AAAATCTATGGGGCTGGGTC	59	461
		*Sph-rbcLX–*R	ACTATTTGGTTTTTGGCACTTA		
*Cuspidothrix*	*rpoB*	*Cus-rpoB–*F	TCGCCTATTCTCACCAATGG	58	496
		*Cus-rpoB*–R	ATCAAAGGTCCACAAGTACC		
*Raphidiopsis*	*rpoC1*	*Raphi-rpoC1*-F	TACCCTCAAGCCAGAAATGG	55	353
		*Rahpi-rpoC1-*R	TGGTCTTCTGTTAATAACTGC		
*Cylindrospermopsis*	*rpoC1*	*Cyl-rpoC1-*F	ATTTTGTGAGCGGATCTTTG	55	325
		*Cyl-rpoC1-*R	GGTCTTCTGTTAACAGTTGT		
*Chrysosporum*	*cpcBA*	*Chry-cpcBA-*F	TTGAACGGTTTGCGCGAAACC	58	449
		*Chry-cpcBA-*R	ACAGCTTCGGTTGCACCATCAATT		

**Table 4:** Community structure of the phytoplankton in field samples (cells·mL^−1^); cell densities of total phytoplankton and major groups (upper) and cyanobacterial genera (bottom).

**No.**	**Station**	**Sampling Date**		**Total Phytoplankton**		**Diatoms**		**Green Algae**		**Other Flagellates**	**Cyanobacteria**
1	SJ	19.08.19		40,990		130		6740		2330	31,790
2	ND	19.08.05		98,764		450		7020		420	90,874
3	GM	19.07.08		9677		65		1000		3350	5262
4	GM	19.08.05		21,074		88		2665		159	18,162
5	CG	19.07.29		18,464		2850		820		3380	11,414
6	CG	19.08.26		15,408		920		490		1600	12,398
7	CG	19.09.30		19,933		5700		3140		9800	1293
8	DS	19.08.19		31,653		1360		3350		12,220	14,723
9	DS	19.09.09		48,858		13,920		4860		1700	28,378
10	DS	19.09.26		2761		1132		732		314	583
11	HC	19.07.08		326,528		215		2675		595	323,043
12	CH	19.09.16		89,470		5470		1990		2280	79,730
13	CH	19.09.30		28,935		12,600		1280		10,640	4415
**No.**	***Microsystis* spp.**		***Aphanizomenon* spp.**		***Dolichospermum* spp.**		***Cylindrosper-mopsis* spp.**		***Cuspidothrix* spp.**		***Sphaerospermopsis* spp.**
1	8160		0		0		0		0		0
2	36,350		534		28,112		0		1937		308
3	1025		100		2503		0		254		0
4	11,810		169		3469		0		755		25
5	1765		0		0		0		39		0
6	7110		30		0		0		0		88
7	755		41		0		77		120		0
8	7335		5435		964		0		29		0
9	22,380		0		10		0		0		48
10	350		30		18		23		0		25
11	258,800		4624		179		0		0		0
12	65,500		595		1122		0		170		103
13	1125		239		153		0		21		0

## Supplementary Materials

The following are available online at https://www.mdpi.com/article/10.3390/ijerph18115703/s1, Figure S1: The results of multiple-sequence comparative analysis of *rbcLX* gene sequences from the nostocalean cyanobacteria; (a) forward, (b) reverse sequence, Figure S2: The results of multiple-sequence comparative analysis of *rpoB* gene sequences from the nostocalean cyanobacteria; (a) forward, (b) reverse sequence, Figure S3: The results of multiple-sequence comparative analysis of *rpoC1* gene sequences from the nostocalean cyanobacteria; (a) forward, (b) reverse sequence, Figure S4: The results of multiple-sequence comparative analysis of *cpcBA* gene sequences from the nostocalean cyanobacteria; (a) forward, (b) reverse sequence.
