# Supplementary material for: Development of Genus-Specific PCR Primers for Molecular Monitoring of Invasive Nostocalean Cyanobacteria
Source: Int J Environ Res Public Health. 2021 May 26;18(11):5703. doi: 10.3390/ijerph18115703 (PMC8198022; doi:10.3390/ijerph18115703)
Supplement: Supplementary file 1 [file ijerph-18-05703-s001.zip › ijerph-1150153-supplementary.pdf]

# Supplementary Figures for Development of Genus-Specific PCR Primers for Molecular Monitoring of Invasive Nostocalean Cyanobacteria

In-Soo Kim[a] , Hae-Kyung Park[a*], Yong-Jin Kim[a]

[a] Nakdong River Environment Research Center, National Institute of Environmental Research, Daegu, Republic of Korea

* Corresponding author : Hae-Kyung Park, parkhknier@korea.kr

Supplementary Figures contain:

5 pages

4 figures

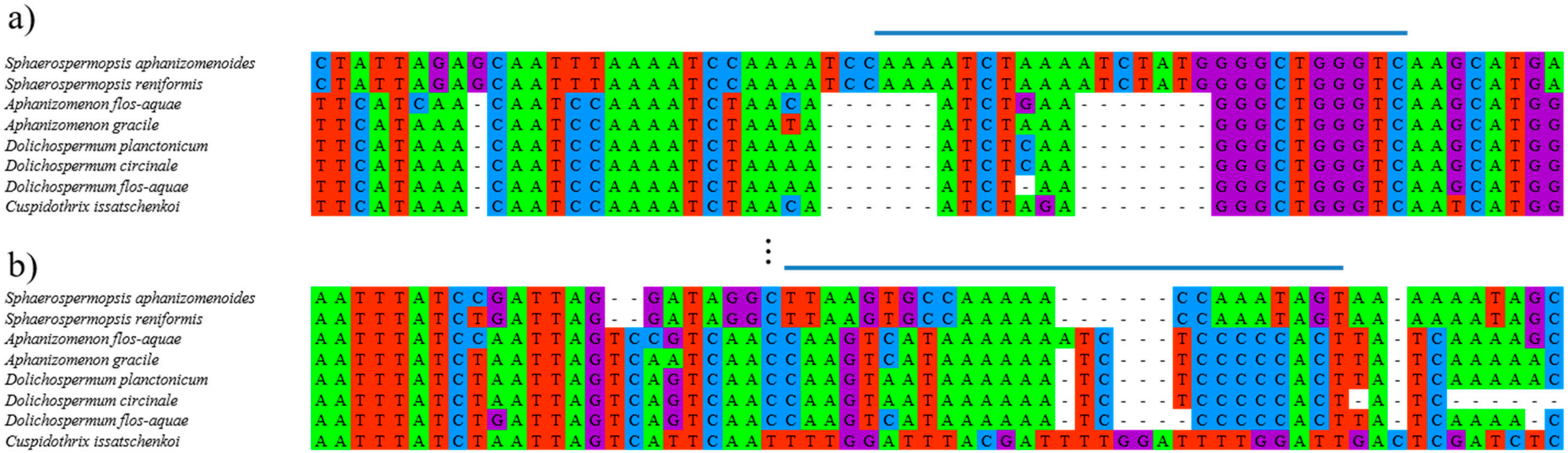


Figure S1. The results of multiple-sequence comparative analysis of *rbcLX* gene sequences from the nostocalean cyanobacteria; a) forward, b) reverse sequence.

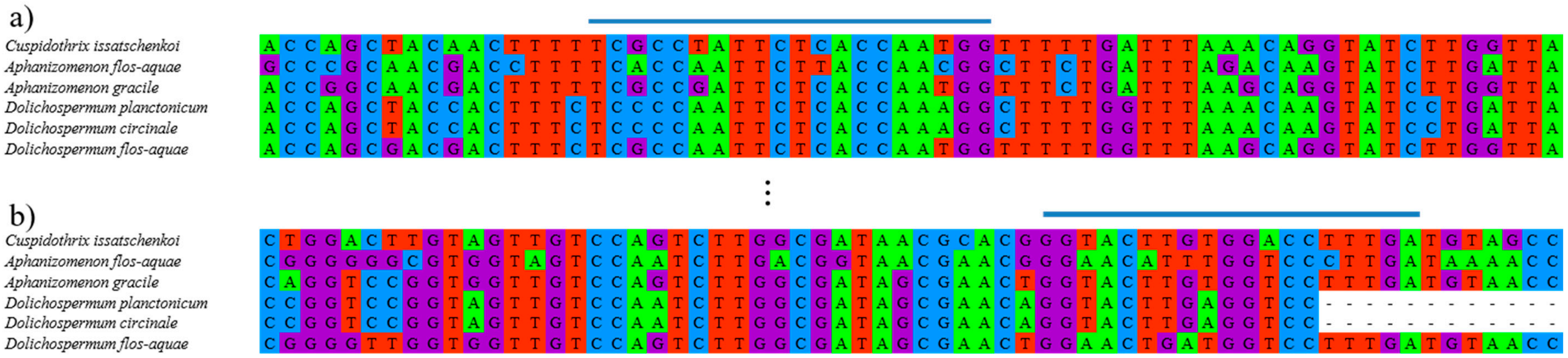


Figure S2. The results of multiple-sequence comparative analysis of *rpoB* gene sequences from the nostocalean cyanobacteria; a) forward, b) reverse sequence.

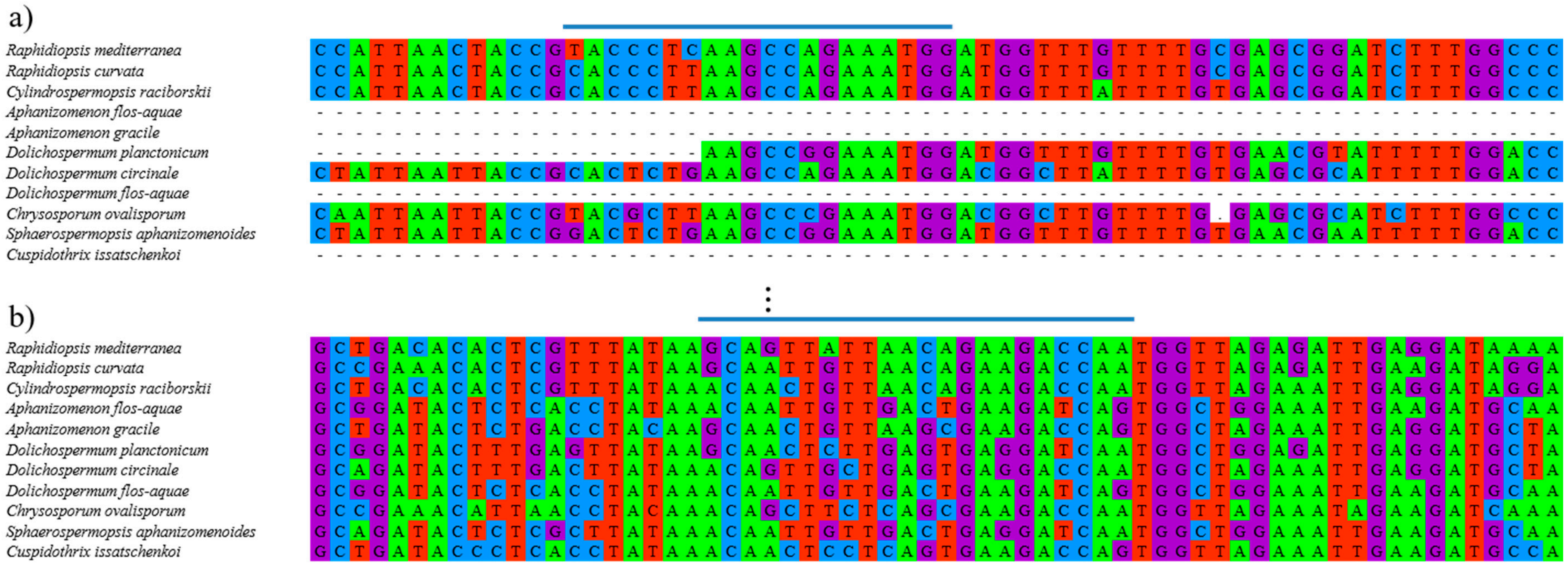


Figure S3. The results of multiple-sequence comparative analysis of *rpoC1* gene sequences from the nostocalean cyanobacteria; a) forward, b) reverse sequence.

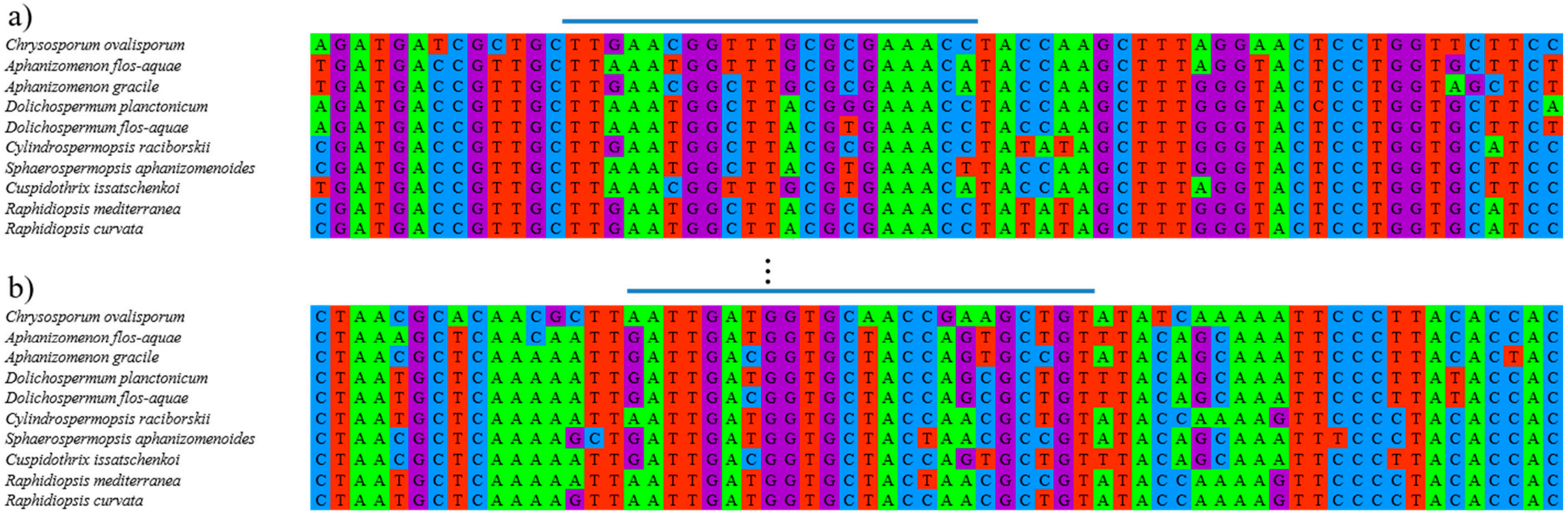


Figure S4. The results of multiple-sequence comparative analysis of *cpcBA* gene sequences from the nostocalean cyanobacteria; a) forward, b) reverse sequence.
